# Differences in breast and cervical cancer screening between West and East Germany: a secondary analysis of a german nationwide health survey

**DOI:** 10.1186/s12889-023-16849-4

**Published:** 2023-10-05

**Authors:** Lena Marie Großmann, Hendrik Napierala, Wolfram J. Herrmann

**Affiliations:** Institute of General Practice and Family Medicine, Charité - Universitätsmedizin Berlin, Charitéplatz 1, 10117 Berlin, Germany

**Keywords:** Cancer screening, Geographic variations, Participation, Mammography, Pap-test, East/West-Germany

## Abstract

**Background:**

Breast cancer and cervical cancer are among the most common cancers in women in Germany. Early detection examinations such as mammography and the cervical smear test (Pap-test) have been shown to contribute to the reduction in the mortality and/or incidence of these cancers and can be utilised free of charge by women in certain age groups as part of national screening programmes. Analyses show that the use of health services varies regionally, especially when comparing the federal states of the former German Democratic Republic (GDR, Eastern Germany) and the Federal Republic of Germany (FRG, Western Germany). This study investigated to what extent the utilisation of mammography examinations and Pap-tests by women differs in federal states of former GDR and FRG.

**Methods:**

For this purpose, we analysed data from the nationwide health survey GEDA14/15 conducted by the Robert Koch Institute (RKI) in 2014 and 2015. We calculated weighted proportions and compared attendance between eastern and western German states by a Chi-Square-test. Additionally, we conducted regression analysis to adjust for socio-economic status, living environment and place of birth.

**Results:**

2,772 female participants aged 20–34 years were analysed for Pap-test attendance in the last two years and 4,323 female participants aged 50–69 years old were analysed for mammography screening attendance in the last two years. 50–69-year-old women in eastern German states were with 78.3% (95%-CI 75.3%, 81.2%) more likely to attend mammography screening than in western Germany with 73.4% (95%-CI 71.8%, 74.9%). Pap-test uptake was statistically significantly higher in the East of Germany with 83.3% (95%-CI 79.6%, 87.1%) compared to 77.5% (95%-CI 75.8%, 79.3%) in the West of Germany. This relationship was robust to adjusting for socio-economic status, living environment and place of birth.

**Conclusions:**

Cultural influences and socialization in the GDR might explain the higher utilisation of these cancer screening examinations at least to some extent. This could have many reasons, for example a higher health awareness through education or a possible greater trust in medical structures and the associated higher compliance of women. These hypotheses should be further explored to increase the uptake of screening examinations by women in Germany.

## Background

Cancer screening is an integral part of public health in most parts of the world. The public health aim of cancer screening is to detect early stages of the cancer to reduce morbidity and mortality. For women, mammography as breast cancer screening and Pap-test as cervical cancer screening have been implemented in many countries worldwide [1].

### Mammography

With an estimated 2.1 million new cases per year worldwide and around 70.000 new cases per year in Germany, breast cancer is the most frequent malignancy in women [2]. In Germany, one in eight women will develop breast cancer during their lives [3]. The greatest population-related risk factor for the development of breast cancer is advanced age. For the early detection of breast cancer, mammography is the only method with a proven reduction in breast cancer mortality [4–6]. Despite the risk of radiation exposure and possible false positive results a nationwide mammography screening program was designed in 2002 according to the EU guidelines [7] and implemented nationwide in Germany by 2009. Within the framework of the program, all women aged 50 to 69 who reside in Germany are entitled to a mammography examination every two years [7]. Eligible women are invited by central offices, which obtain the relevant addresses from the residents’ registration offices. In 2019, 96% of all eligible women were invited to the screening program [8]. Of these, around 50% of the invited women participated in the mammography screening programme [8].

### Pap-test

Cervical cancer is the third most common cancer in women and are mostly caused by prior human papillomavirus infections [9]. In 1971, a nationwide screening scheme for cervical cancer was introduced in Germany. Under this scheme, women aged 20 and over are entitled to an annual genital examination, a medical history interview about risk factors and a Pap-test [10]. The Pap-test involves taking cells from the surface and cervical canal with a brush, fixation and staining of these cells on a microscope slide and observing and assessing the morphology of the cells, their hormone status, and signs of infections or degeneration. With the introduction of the screening scheme the incidence of invasive cervical carcinoma in Germany fell sharply. This decline is attributed to the screening programme [9]. In its current version (2020), women aged 35 and over also receive an HPV test every three years.

Utilisation of health services in eastern and western Germany.

The utilisation of cancer screening varies regionally [11]. In general, the utilization of preventive health services such as cancer screening is influenced by individual and environmental factors. Known individual factors for the utilization of breast cancer screening are age and education and for cervical cancer screening education and marital status [12]. Additionally, there are environmental factors such as the regional availability of health services [13].

From 1945 to 1990 Germany had been split into two separate parts, the Federal Republic of Germany (FRG) in the West and the German Democratic Republic (GDR) in the East. While the health care system in West Germany followed the Bismarckian Model with strong specialist service and weak primary care and public health services, the system in the East emphasized primary care and public health [14]. For example, in the GDR there were regular health check-ups for children, a structured program to combat tuberculosis and compulsory vaccinations for polio.

Research indicates that more than 20 years after the unification of Germany, there remain differences in health care behaviour [13, 15]. This is reflected, among other things, in a better vaccination coverage of older people against influenza in federal states of former GDR compared to the FRG. More recent research, however, increasingly observes a convergence, and in some cases even an equalisation, of the East-West inequalities in health [16].

Thus, our goal was to determine if 25 years after reunification, there are still differences in the uptake of screening examinations for breast cancer and cervical cancer between women living in federal states of former GDR and FGR.

## Methods

To research differences in breast and cervical cancer screening uptake between eastern and western German states, we analysed representative survey data from the nationwide survey “German Health update” (GEDA) from 2014/2015.

### Underlying data from the GEDA 2014/2015 survey

The nationwide survey “German health update” (GEDA) was conducted by the Robert Koch Institute (RKI) on behalf of the Federal Ministry of Health (Bundesministerium für Gesundheit) from November 2014 to July 2015. The survey is part of the health monitoring in Germany and is repeated regularly [17]. Within the scope of this survey, 24,016 persons aged 18 years and older answered questions about their health status, health behaviour and general health care via web or paper questionnaires. The response rate was 26.9%. Individuals were randomly selected from the local population registers of 301 previously randomly selected municipalities representing different regions in Germany. All persons were besides other things asked about their gender, date of birth and country of origin. As potential confounders, we included the socio-economic status [18], the living environment of the participants (rural districts, sparsely populated rural districts, mainly urbanized districts, and larger cities) and the place of birth (inside vs. outside of Germany) into the analysis.

### Statistical evaluation

The statistical analysis was carried out in “R”, using the “Survey”-package [19, 20] to take into account the design effects of the survey by using the weights provided by the RKI.

The following variables were considered for this work: As eligibility criteria we used age (in 5- and 10-year increments), sex (male, female) and the information on the time of the last mammography examination or the last Pap-test performed.

For the analysis of the utilisation of mammography examinations, all women aged 50 to 69 years were evaluated as eligible. Eligible women who, according to self-report, had undergone a mammography examination within the last two years as recommended were counted as attenders in the evaluation. All types of reported mammography were included no matter if as part of the screening program or for diagnostic/curative purposes. The proportion of women who attended mammography screening was calculated from the eligible women on the national level, on level of the federal state, and separately for eastern and western Germany, whereby Berlin was considered a separate category due to its special historical position.

The Chi-squared test for independent categorical variables was used to test whether the difference in the participation rate in the mammography screening programme between eastern and western Germany was statistically significant. Berlin was excluded from the analysis. The significance level was set at α = 0.05. The calculation of the 95%-confidence intervals was carried out by means of non-parametric bootstrapping using the “boot” package [21] in 10,000 replicates using the weights provided.

The calculation of the uptake of the Pap-test was carried out analogously to the analysis of the mammography uptake on the national level, for each federal state as well as in the east-west comparison. Women between 20 and 34 years of age were considered eligible, as an annual cervical smear test is required in this age group. Women of this age who had a Pap-test within the last two years were counted as attenders.

To adjust for potential confounders, we conducted weighted logistic regressions with the covariates socio-economic status, living environment and place of birth.

## Results

Of the respondents, 13,144 were women, of whom 2,772 were in the age group 20–34 years old; 118 of these lived in Berlin, 594 in the federal states of former GDR and 2060 in the federal states of former FRG. 4,323 women were in the age group 50–69 years old; 129 of these lived in Berlin, 960 in the federal states of former GDR and 3,234 in the federal states of former FRG.

### Utilisation of the mammography examination

**Fig. 1:**
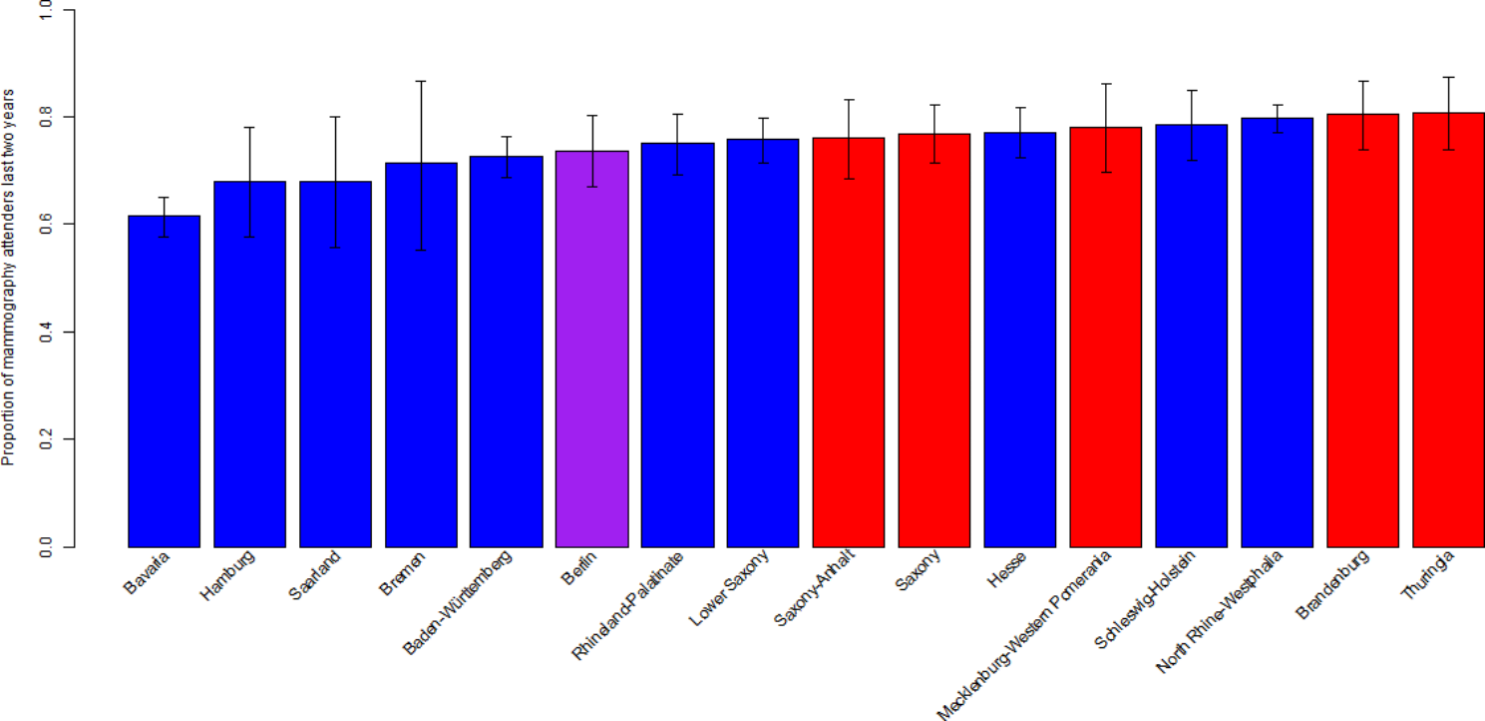
Proportion of mammography attenders in the age of 50–69 in Germany by federal state (blue former FRG, red former GDR, purple Berlin) with 95%-CI

3,152 of the participating women aged 50–69 at the time of the survey had had a mammography examination in the last two years. This corresponds to a population-weighted ratio of 74.2% (95%-CI 72.9%,75.6%) mammography attenders in women aged 50–69 in Germany. The mammography attendance rate was highest in Thuringia with 80.8% (95%-CI 73.9%,87.6%) and lowest in Bavaria with 61.5% (95%-CI 57.7%,65.2%; cf. Figure 1). There was a statistically significant difference in the attendance rate between 78.3% (95%-KI 75.3%, 81.2%) in eastern Germany and 73.4% (95%-KI 71.8%, 74.9%) in western Germany (p = 0.017; F = 5.80).

### Utilisation of the Pap-test

**Fig. 2:**
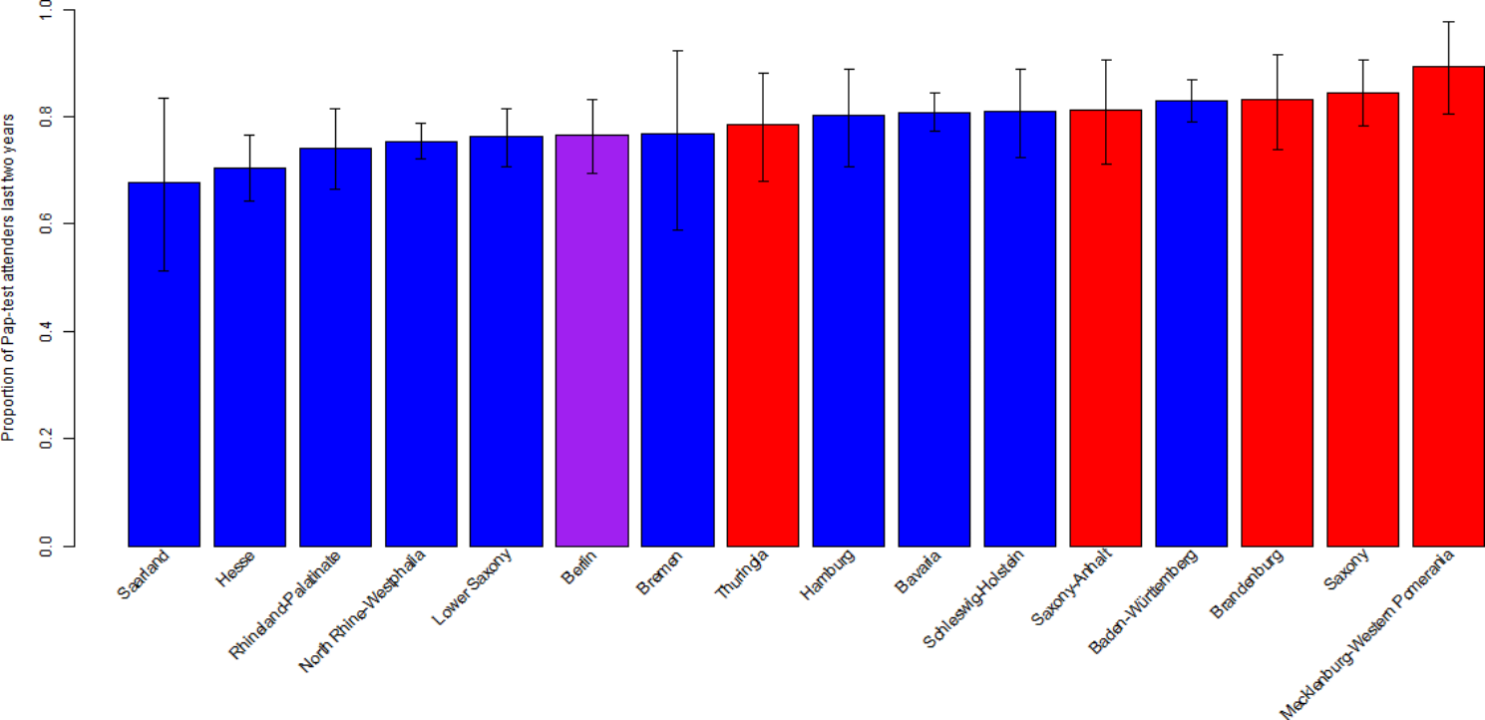
Proportion of Pap-test attenders in the age of 20–34 in Germany by federal state (blue former FRG, red former GDR, purple Berlin) with 95%-CI

2163 of the participating women aged 20–34 years at the time of the survey had had a Pap-Test in the two years before the survey. This corresponds to a population weighted ratio of 78.3% (95%-CI: 76.7%, 79.8%) of women in Germany aged 20 to 34 to have attended a Pap-test in the last two years. The uptake of the Pap-test was highest in Mecklenburg-Western Pomerania with 89.5% (95%-CI 80.0%,97.5%) and lowest with 67.6% (95%-CI 51.5%,82.2%) in Saarland (cf. Figure 2). Pap-test uptake was statistically significantly higher in the East of Germany with 83.3% (95%-CI 79.6%, 87.1%) compared to 77.5% (95%-CI 75.8%, 79.3%) in the West of Germany (p = 0.011, F = 6.63).

In the logistic regression modelling for mammography attendance (cf. Table 1), taking the socio-economic status, living environment and if the participants were born in Germany as potential confounders into the model, living in an eastern state still increased the odds for mammography attendance by 1.44. Modelling Pap-test attendance, adjusting for socio-economic status, living environment, and being born in Germany, living in eastern Germany still increased the Odds for Pap-test attendance by 1.36.

**Table 1:** Odds ratio estimates from the weighted logistic regression on mammography attendance and Pap-test attendance

	Mammography attendance	Pap-test attendance
Living in an eastern federal state	1.44 (1.10,1.89)	1.36 (1.01,1.84)
SES-Score	1.01 (0.99,1.03)	1.09 (1.05,1.13)
Sparsely populated rural districts (vs. rural districts)	0.83 (0.57,1.21)	1.14 (0.71,1.83)
Mainly urbanized districts (vs. rural districts)	1.12 (0.81,1.54)	0.79 (0.55,1.14)
Larger cities (vs. rural districts)	0.98 (0.70,1.37)	0.75 (0.53,1.08)
Born outside Germany	0.88 (0.63,1.22)	0.94 (0.65,1.36)

## Discussion

### Summary

In summary, this study shows that the uptake of mammography and Pap-tests by women in 2014/15 was significantly higher in eastern Germany than in western Germany. At the same time, the participation rate within the eastern and western federal states varied widely. This relationship was robust in regression analysis adjusting for socio-economic status, living environment, and being born in or outside Germany.

### Context of other work

These results partly contradict the RKI’s 2009 report on the development of health in eastern and western Germany [15]. The report assumes that the differences in the use of cancer screening between the federal states of former GDR and FRG that existed at the time of reunification no longer existed in 2009. The report and the results of this study agree that regional fluctuations in the participation rate were observed. However, the report did not look for specific screening measures thus, potentially underestimating the effect.

The statistics regarding nationwide mammography examination uptake can vary depending on the study. These figures, including diagnostic mammography, range from 50% [8] to 74.2% [22]. This is partly due to whether the reason for the mammography was taken into account. In some studies, for example, only women are counted who had a mammography examination as part of the screening process, not to clarify abnormal findings. In this study, the reason for the mammography was not assessed on such detailed level and all women were included who had a mammography in the period mentioned. As a result, the participation rates mentioned in this study could be higher than in other studies.

A systematic review by Dreier et al. [12] assumes a nationwide uptake of the Pap-test of 18–83%. However, public health insurance records indicate that women in their mid-twenties to mid-thirties have shown an uptake of more than 50% [23]. The uptake rates of the Pap-test in this paper may be higher than in others, as only women aged 20–34 were considered. Research suggests that uptake is higher in this age group than in older women [12].

One possible reason for the higher use of mammography and Pap-tests in the federal states of former GDR could be the socialisation of women in the GDR. In the GDR, health care and prevention were seen more as a state task than in western Germany and were organised centrally. However, the thesis of socialisation by the GDR does not justify the contradictory performance of Thuringia in mammography (highest participation rate) and Pap-test (lowest participation rate within eastern federal states). An explanation could be the different coverage with gynaecology practices/mammography centres in the different federal states. For this reason, it is noteworthy that the uptake of the Pap-test is higher in the eastern states, although the density of gynaecologists (where the Pap-test is usually performed) is higher in the western states (16.25 gynaecologists per 100,000 inhabitants) than in the eastern states (14.26 gynaecologists per 100,000 inhabitants). This is contradicted by the fact that the city states with the highest density of gynaecologists (Berlin: 19.3, Hamburg: 21.5, Bremen: 21.6 gynaecologists per 100,000 inhabitants) do not have much higher uptake of Pap-tests. We know that long distances and poor accessibility have a negative impact on the uptake of cancer screening [24]. Therefore, travel and regional infrastructure could also play a role and vary between the different federal states. This thesis is also supported by a study by Vogt et al. [13], where it is stated, that factors such as the accessibility of health care services explain a significant part of the regional variation in the use of cancer screening services, even after controlling for socioeconomic and other regional covariates. Also, they found out, that these rates are clustered regionally due to spill over effects from informal communication and observational learning. Differences between eastern and western Germany were not the central question of the study, but in their results, they mention a higher uptake rate of different cancer screening services in eastern vs. western Germany.

### Limitations

A limitation of this work is that the underlying data had been acquired in 2014/2015 and the difference between eastern and western Germany might meanwhile have been decreasing. Since January 2020, an organised screening program for cervical cancer was introduced that might further reduce the differences. Thus, the difference should be examined with newer data to determine trends. However, the latest survey data are not available to the public yet. The underlying data of this paper is based on women’s self-report, which could bias the results as knowledge and education might influence both the uptake of Pap-test and mammography and the health literacy to answer the question correctly. So it could be that, for example, more women took a Pap-test than was reported because they simply did not know what the routine test was about. Furthermore, with the low response rate in this survey, a response bias could exist since participation in the survey is voluntary and possibly health-conscious people participated more often in the survey. The women were divided into East and West according to their current reported place of residence. However, this did not take into account where they actually grew up beyond inside and outside Germany.

## Conclusion

The utilisation of cancer screening examinations by women in Germany varies considerably from region to region. A significantly higher uptake of mammography examinations and Pap-tests was observed in the eastern federal states which belonged formerly to the GDR compared to the western federal states which belonged formerly to the FRG. These results persist also after regression analysis adjusting for socio-economic status, living environment and place of birth. Thus, we can assume that socialisation in former eastern or western Germany plays a role in the uptake of cancer screening examinations. This highlights the influence of enduring traits on healthcare utilization behaviour, which helps explain why interventions aimed at enhancing uptake often fall short of achieving the expected significant results.

## Data Availability

A public use file of the survey is available upon request from the Robert-Koch-Institute: https://www.rki.de/EN/Content/Health_Monitoring/Public_Use_Files/public_use_file_node.html The data sharing agreement does not allow to share the data directly.

## Abbreviations

GDR: German Democratic Republic
FRG: Federal Republic of Germany
GEDA14/15: Gesundheit in Deutschland aktuell / German Health Update Survey
RKI: Robert Koch Institute
CI: Confidence Interval
HPV: Human Papillomavirus
